# An anti-TROP2 monoclonal antibody TrMab-6 exerts antitumor activity in breast cancer mouse xenograft models

**DOI:** 10.3892/or.2021.8083

**Published:** 2021-05-19

**Authors:** Tomohiro Tanaka, Tomokazu Ohishi, Teizo Asano, Junko Takei, Ren Nanamiya, Hideki Hosono, Masato Sano, Hiroyuki Harada, Manabu Kawada, Mika K. Kaneko, Yukinari Kato

**Affiliations:** 1 Department of Antibody Drug Development, Tohoku University Graduate School of Medicine, Aoba-ku, Sendai, Miyagi 980-8575, Japan; 2 Institute of Microbial Chemistry (BIKAKEN), Numazu, Microbial Chemistry Research Foundation, Numazu-shi, Shizuoka 410-0301, Japan; 3 Department of Oral and Maxillofacial Surgery, Graduate School of Medical and Dental Sciences, Tokyo Medical and Dental University, Bunkyo-ku, Tokyo 113-8510, Japan; 4 New Industry Creation Hatchery Center, Tohoku University, Aoba-ku, Sendai, Miyagi 980-8575, Japan

**Keywords:** TROP2, monoclonal antibody, ADCC, CDC, antitumor activity, breast cancer

## Abstract

Trophoblast cell surface antigen 2 (TROP2), reported to be overexpressed in several types of cancer, is involved in cell proliferation, invasion, metastasis, and poor prognosis of many types of cancer. Previously, a highly sensitive anti-TROP2 monoclonal antibody (clone TrMab-6; mouse IgG_2b_, κ) was developed using a Cell-Based Immunization and Screening (CBIS) method. TrMab-6 was useful for investigations using flow cytometry, western blot, and immunohistochemistry. The aim of the present study was to investigate whether TrMab-6 possesses *in vitro* antibody-dependent cellular cytotoxicity (ADCC) or complement-dependent cytotoxicity (CDC) activities or *in vivo* antitumor activities using mouse xenograft models of TROP2-overexpressed CHO-K1 (CHO/TROP2) and breast cancer cell lines, including MCF7, MDA-MB-231, and MDA-MB-468. *In vitro* experiments revealed that TrMab-6 strongly induced ADCC and CDC activities against CHO/TROP2 and the three breast cancer cell lines, whereas it did not show those activities against parental CHO-K1 and MCF7/TROP2-knockout cells. Furthermore, *in vivo* experiments on CHO/TROP2 and MCF7 ×enografts revealed that TrMab-6 significantly reduced tumor growth, whereas it did not show antitumor activities against parental CHO-K1 and MCF7/TROP2-knockout xenografts. The findings suggest that TrMab-6 is a promising treatment option for TROP2-expressing breast cancers.

## Introduction

The loss of epithelial features in tumors, known as epithelial-mesenchymal transition (EMT), is significantly involved in the malignant transformation of cancers, such as tumor initiation, migration, and metastasis (1,2). Previous findings have identified several molecules associated with the maintenance of the epithelial features of cells (3). Epithelial cell adhesion molecule (EpCAM) is a cell adhesion transmembrane molecule, which is overexpressed in tumors. EpCAM is also known as trophoblast cell surface antigen 1 (TROP1) and it is encoded by the tumor-associated calcium signal transducer 1 (*TACSTD1*) gene (3). Trophoblast cell surface antigen 2 (*TROP2*), another molecule of the *TACSTD* gene family, was identified as a cell surface marker for invasive trophoblast cells (4). TROP2 is a promising therapeutic target (5), and its expression is associated with cancer malignancy in various solid tumors including breast cancers (6).

TROP2 is a 46-kDa type I transmembrane glycoprotein (323 amino acids), which consists of a large extracellular domain (274 amino acids) with four *N*-glycosylation sites, a transmembrane domain (23 amino acids), and a short intracellular domain (26 amino acids). TROP2 possesses 49% identity and 67% similarity with EpCAM (4,5), and is expressed in normal tissues, such as skin, kidney, liver, breast, ureteric bud, and renal tubules. TROP2 is highly expressed during the development of mammalian embryos and fetus (4,7,8).

TROP2 has been reported to be overexpressed in cancers, and is involved in cell proliferation, invasion, metastasis, and poor prognosis in many cancer types (9–12). It has been reported that membrane-localized TROP2 becomes an unfavorable target of prognosis, while the intracellular retention of TROP2 is associated with less frequent tumor relapse and better survival in breast cancer patients (13). A high expression of TROP2 and low expression of E-cadherin are associated with lymph node status, metastasis, tumor/node/metastasis (TNM) stage, and ER/PR/HER2 expression, indicating that TROP2 is considered to have a potential role in the promotion of EMT (14). Furthermore, TROP2 has been reported to be involved in the chemotherapeutic resistance against lung cancer (15).

Previously, we developed a highly sensitive anti-TROP2 monoclonal antibody (mAb; clone TrMab-6; mouse IgG_2b_, kappa) (16) using a Cell-Based Immunization and Screening (CBIS) method (17). TrMab-6 was useful for investigations using flow cytometry, western blot, and immunohistochemistry (16). The aim of this study was to investigate whether TrMab-6 possesses *in vitro* antibody-dependent cellular cytotoxicity (ADCC) or complement-dependent cytotoxicity (CDC) activities and *in vivo* antitumor activities using breast cancer models.

## Materials and methods

### 

#### Cell lines

CHO-K1 and the breast cancer cell lines, MDA-MB-231 and MDA-MB-468 were obtained from the American Type Culture Collection. The breast cancer cell line MCF7 was obtained from the Cell Resource Center for Biomedical Research Institute of Development, Aging and Cancer, at Tohoku University, Japan. C-terminal PA-tagged TROP2-overexpressed CHO-K1 (CHO/TROP2) was previously established by transfection of pCAG/TROP2-PA to CHO-K1 cells using Lipofectamine LTX Reagent (Thermo Fisher Scientific, Inc.) (16). The *TROP2* gene-knockout cell line, MCF7/TROP2-KO (BINDS-29), was previously generated by transfection of CRISPR/Cas9 plasmids targeting TROP2 (http://www.med-tohoku-antibody.com/topics/001_paper_cell.htm), using the Neon Transfection System (Thermo Fisher Scientific, Inc.). Stable transfectants were established by cell sorting using SH800 (Sony Biotechnology Corp.) (16). CHO-K1, CHO/TROP2, MCF7, and BINDS-29 were cultured in Roswell Park Memorial Institute (RPMI)-1640 medium (Nacalai Tesque, Inc.). MDA-MB-231 and MDA-MB-468 were cultured in Dulbecco's modified Eagle's medium (DMEM; Nacalai Tesque, Inc.). RPMI-1640 and DMEM were supplemented with 10% heat-inactivated fetal bovine serum (FBS; Thermo Fisher Scientific Inc.), 100 U/ml of penicillin (Nacalai Tesque, Inc.), 100 µg/ml streptomycin (Nacalai Tesque, Inc.), and 0.25 µg/ml amphotericin B (Nacalai Tesque, Inc.), and incubated at 37°C in a humidified atmosphere containing 5% CO_2_.

#### Primary antibodies

Purified mouse IgG (cat. no. I8765) and mouse IgG_2b_ (cat. no. M1395) were purchased from Sigma-Aldrich; Merck KGaA. An anti-TROP2 mAb was purified using Protein G-Sepharose (GE Healthcare Biosciences).

#### Western blot analysis

Cell pellets were resuspended in phosphate-buffered saline (PBS; Nacalai Tesque, Inc.) with 1% Triton X-100 (cat. no. 168-11805; FUJIFILM Wako Pure Chemical Corporation) and 50 µg/ml aprotinin (product no. 03346-84; Nacalai Tesque, Inc.). Cell debris was removed by centrifugation at 21,880 × g for 10 min at 4°C. Protein concentration was determined by BCA method. Cell lysates were boiled in sodium dodecyl sulfate sample buffer with a reducing reagent (Nacalai Tesque, Inc.). These proteins (10 µg) were electrophoresed on 5–20% polyacrylamide gels (FUJIFILM Wako Pure Chemical Corporation) and transferred onto polyvinylidene difluoride (PVDF) membranes (Merck KGaA). After blocking with 4% skim milk (Nacalai Tesque, Inc.) at room temperature for 30 min, the membranes were incubated with primary antibodies, such as 1 µg/ml of TrMab-6 or anti-β-actin for control (clone AC-15; Sigma-Aldrich; Merck KGaA) at room temperature for 30 min, followed by incubation with secondary peroxidase-conjugated anti-mouse immunoglobulins (1:1,000; cat. no. P044701-2; Agilent Technologies Inc.) at room temperature for 30 min. Finally, the proteins were visualized with ImmunoStar LD (cat. no. 290-69904; FUJIFILM Wako Pure Chemical Corporation) or Pierce™ ECL Plus Western Blotting Substrate (cat. no. 32132; Thermo Fisher Scientific, Inc.), and were detected using the Sayaca-Imager (DRC Co. Ltd.). Qcapture Pro software (DRC Co. Ltd) was used for the densitometry.

#### Flow cytometry

Cells (2×10^5^ cells/ml) were harvested after brief exposure to 0.25% trypsin in 1 mM ethylenediaminetetraacetic acid (EDTA; Nacalai Tesque, Inc.). After washing with 0.1% bovine serum albumin (BSA, Nacalai Tesque, Inc.) in PBS the cells were treated with 1 µg/ml of an anti-TROP2 mAb or control (1% BSA in PBS; blocking buffer) for 30 min at 4°C, and then with Alexa Fluor 488-conjugated anti-mouse IgG (1:1,000; product no. 4408; Cell Signaling Technology, Inc.). Fluorescence data were collected using flow cytometer: SA3800 Cell Analyzer (Sony Biotechnology Corp.).

#### ADCC

ADCC stimulation by an anti-TROP2 mAb was assayed as follows. Five female five-week-old BALB/c nude mice (mean weight, 15±3 g) were purchased from Charles River Laboratories, Inc. Mice were kept under specific pathogen-free conditions on an 11-h light/13-h dark cycle at a temperature of 23±2°C and 55±5% humidity with food and water supplied *ad libitum* during the experimental periods. After euthanasia by cervical dislocation, spleens were removed aseptically, and single-cell suspensions were obtained by forcing spleen tissues through a sterile cell strainer (product no. 352360; Corning, Inc.) with a syringe. Erythrocytes were lysed with 10-sec exposure to ice-cold distilled water. The splenocytes were then washed with DMEM and resuspended in DMEM with 10% FBS; this preparation was designated as effector cells. The target tumor cells were labeled with 10 µg/ml Calcein-AM (Thermo Fisher Scientific, Inc.) and resuspended in the same medium. The target cells were transferred to 96-well plates, at 2×10^4^ cells/well, and mixed with effector cells at an effector-to-target ratio of 100:1, along with 100 µg/ml of an anti-TROP2 mAb or control mouse IgG_2b_. After a 5-h incubation at 37°C, the Calcein-AM release into the supernatant was measured for each well. Fluorescence intensity was assessed using a microplate reader (Power Scan HT; BioTek Instruments, Inc.) with an excitation wavelength of 485 nm and an emission wavelength of 538 nm. Cytotoxicity (as % lysis) was measured using the formula: Percentage of lysis (%)=(E-S)/(M-S) ×100, where E is the fluorescence released in combined cultures of target cells and effector cells, S is the spontaneous fluorescence released in cultures of only target cells, and M is the maximum fluorescence measured after lysis of all cells with buffer containing 0.5% Triton X-100, 10 mM Tris-HCl (pH 7.4), and 10 mM EDTA. Animal studies for ADCC and the antitumor activity were approved by the Institutional Committee for experiments of the Institute of Microbial Chemistry (permit no. 2020-015).

#### CDC

CDC stimulation by an anti-TROP2 mAb was assayed as follows. Target cells were labeled with 10 µg/ml Calcein-AM (Thermo Fisher Scientific, Inc.) and resuspended in medium. Target cells were plated in 96-well plates, at 2×10^4^ cells/well, and 10% rabbit complement (Low-Tox-M rabbit complement; Cedarlane Laboratories) and 100 μg/ml of an anti-TROP2 mAb or control IgG (mouse IgG_2b_) were added to each well. After 5 h of incubation at 37°C, the Calcein-AM release into the supernatant was measured for each well. Fluorescence intensity was calculated as described in the ADCC section above.

#### Antitumor activity of an anti-TROP2 mAb in a mouse xenograft model

Sixty-four five-week-old female BALB/c nude mice (mean weight, 15±3 g) were purchased from Charles River Laboratories, Inc., and were divided into the following four groups (n=16 in each group): i) CHO/TROP2-bearing mice, ii) CHO-K1-bearing mice, iii) MCF7-bearing mice, and iv) BINDS-29-bearing mice. On day 7, each group was subdivided into 2 groups (n=8 in each group) with equal mean tumor volume: A control mouse IgG-treated group or an anti-TROP2 mAb-treated group. All animal experiments were performed in accordance with institutional guidelines and regulations to minimize animal suffering and distress in the laboratory. The Institutional Committee for experiments of the Institute of Microbial Chemistry (permit no. 2020-015) approved the animal studies for antitumor activity.

Mice were maintained in a pathogen-free environment, on an 11-h light/13-h dark cycle at a temperature of 23±2°C and 55±5% humidity, with food and water supplied *ad libitum* throughout the experiments. Mice were monitored for health and weight every three or four days. Experiments on mice were conducted in four weeks. Weight loss >25% or tumor volume >3,000 mm^3^ was identified as humane endpoints for euthanasia. At humane and experimental endpoints, mice were euthanized by cervical dislocation, and death was verified by validating respiratory and cardiac arrest.

After an acclimation period of one week, these mice were used in experiments at six weeks of age (mean weight, 16±2 g). Cells (0.3 ml of 1.33×10^8^ cells/ml in DMEM) were mixed with 0.5 ml BD Matrigel Matrix Growth Factor Reduced (BD Biosciences). A total of 100 µl of this suspension (5×10^6^ cells) was injected subcutaneously into the left flank of each animal. On day 7 post-inoculation, 100 µg of an anti-TROP2 mAb or control mouse IgG in 100 µl PBS was injected intraperitoneally (i.p.). Additional antibody inoculations were performed on days 14 and 21. Twenty-four days after cell implantation, all mice were euthanized by cervical dislocation, and tumor diameters and volumes were measured and recorded.

#### Statistical analysis

Data are expressed as mean ± standard error of the mean (SEM). Statistical analysis was conducted with Welch's t-test for ADCC and CDC, ANOVA and Sidak's multiple comparisons tests for tumor volume and mouse weight, and Welch's t-test for tumor weight. All calculations were performed using GraphPad Prism 7 (GraphPad Software, Inc.). P<0.05 was considered statistically significant.

## Results

### 

#### Western blot analysis

**Figure 1.**
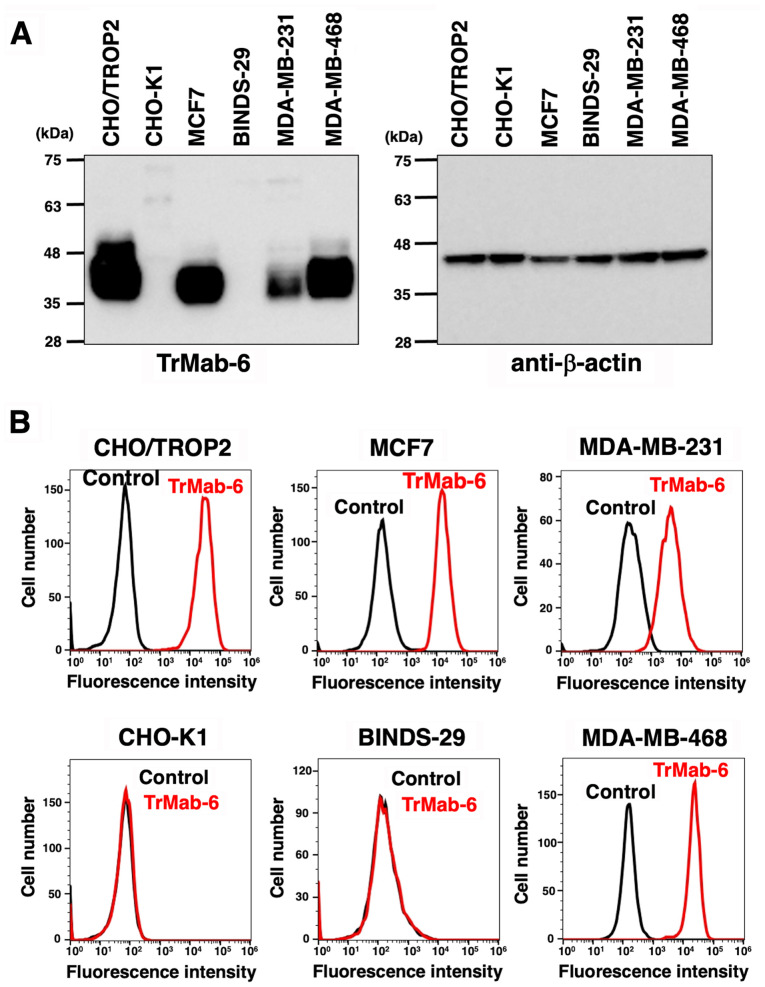
(A) Detection of TROP2 by TrMab-6 by western blot analysis. Cell lysates of CHO/TROP2, CHO-K1, MCF7, BINDS-29, MDA-MB-231, and MDA-MB-468 cells were electrophoresed and transferred onto PVDF membranes. These membranes were treated with TrMab-6 (left panel) or anti-β-actin (right panel), followed by incubation with peroxidase-conjugated anti-mouse immunoglobulin. (B) Flow cytometry using TrMab-6. CHO/TROP2, CHO-K1, MCF7, BINDS-29, MDA-MB-231, and MDA-MB-468 cells were treated with 1 µg/ml of TrMab-6, followed by a treatment with Alexa Fluor 488-conjugated anti-mouse IgG. Black line, negative control (blocking buffer).

We performed western blot analysis using TrMab-6. TrMab-6 detected TROP2 with a 40-kDa band in CHO/TROP2 (16), MCF7, MDA-MB-231, and MDA-MB-468 cells; however, it did not detect any proteins in CHO-K1 and BINDS-29 cells (Fig. 1A), indicating that TrMab-6 is specific for TROP2. As TROP2 is over-expresed in CHO/TROP2, the band in the CHO/TROP2 cell was broader than that of the other cells, such as MCF7, MDA-MB-231, and MDA-MB-468. We used β-actin as an internal control.

#### Flow cytometry

We investigated whether TrMab-6 can react with CHO-K1, CHO/TROP2 (16), MCF7, BINDS-29 (MCF7/TROP2-KO), MDA-MB-231, and MDA-MB-468 by flow cytometry. We used a blocking buffer as negative control. TrMab-6 recognized the CHO/TROP2 cells, but not the parental CHO-K1 cells (Fig. 1B). TrMab-6 also recognized the endogenous TROP2 in MCF7 breast cancer cells (Fig. 1B). By contrast, the reaction of TrMab-6 to BINDS-29 was lost after the knockout of TROP2 in MCF7 cells (Fig. 1B), indicating that TrMab-6 is specific for TROP2. TrMab-6 also detected TROP2 of MDA-MB-231 and MDA-MB-468 (Fig. 1B).

#### ADCC and CDC activities of TrMab-6 in TROP2-expressing cell lines

**Figure 2.**
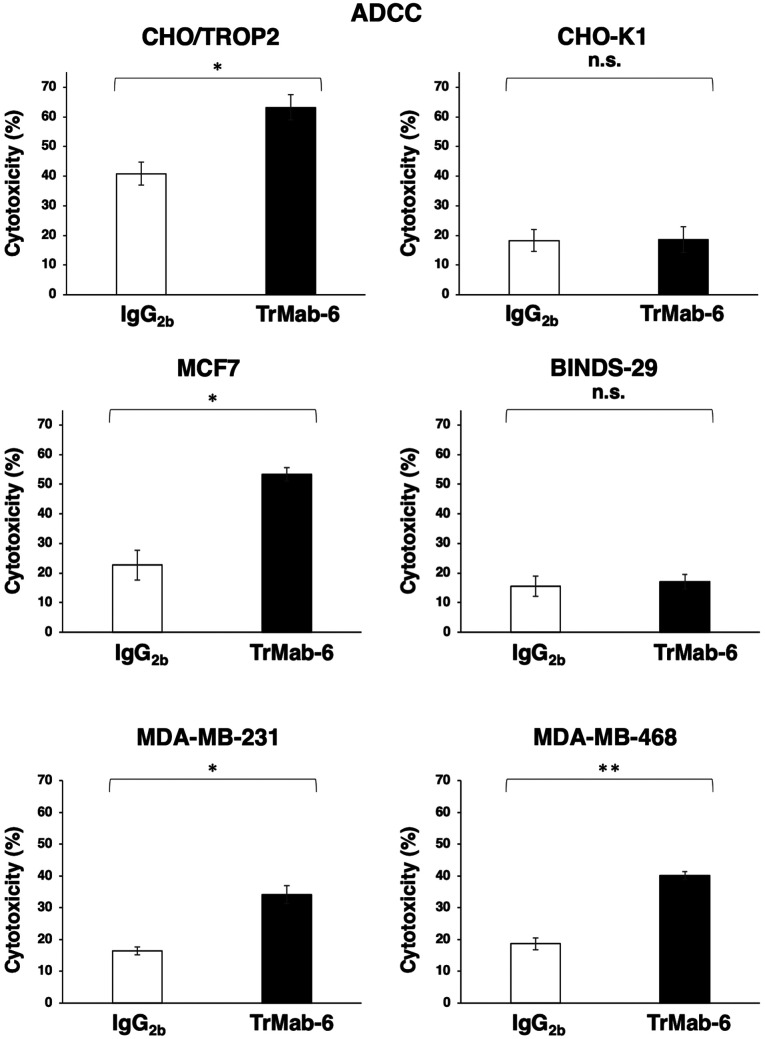
Evaluation of ADCC activities by TrMab-6. ADCC activities by TrMab-6 and control mouse IgG_2b_ in CHO/TROP2, CHO-K1, MCF7, BINDS-29, MDA-MB-231, and MDA-MB-468 cells. The cells were incubated with splenocytes from female nude mice in the presence of the indicated antibodies at 100 µg/ml for 5 h. Cytotoxicity (%) was calculated as described in Materials and methods. Values are mean ± SEM. Asterisk indicates statistical significance (**P<0.01, *P<0.05, n.s., not significant, Welch's t-test).

The effect of TrMab-6 (mouse IgG_2b_) in the ADCC and CDC activity in TROP2-expressing cells, such as CHO/TROP2 (16) or MCF7, MDA-MB-231, and MDA-MB-468 breast cancer cell lines, was analyzed. First, TrMab-6 exhibited higher ADCC (63.2% cytotoxicity) in CHO/TROP2 cells than that of the control mouse IgG_2b_ (40.9% cytotoxicity; P<0.05) (Fig. 2). By contrast, TrMab-6 did not show any ADCC activity in CHO-K1 cells compared with the respective control (Fig. 2). Additionally, TrMab-6 exhibited higher ADCC (53.3% cytotoxicity) in MCF7 cells that in the control mouse IgG_2b_ (22.7% cytotoxicity; P<0.05); however, no ADCC activity was observed in BINDS-29 cells (Fig. 2). TrMab-6 also exhibited higher ADCC (34.2% cytotoxicity) in MDA-MB-231 cells than that of the control mouse IgG_2b_ (16.4% cytotoxicity; P<0.05) (Fig. 2). Furthermore, TrMab-6 exhibited higher ADCC (40.2% cytotoxicity) in MDA-MB-468 cells than that of the control mouse IgG_2b_ (18.7% cytotoxicity; P<0.01) (Fig. 2).

**Figure 3.**
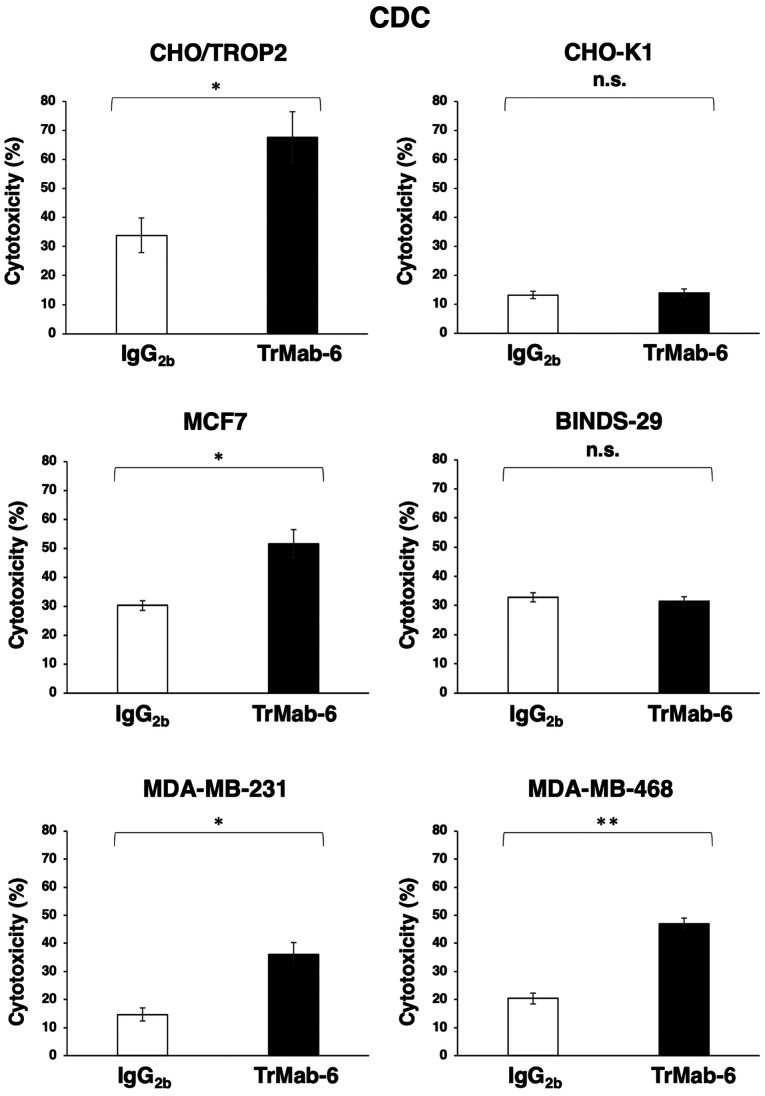
Evaluation of CDC activities by TrMab-6. CDC activities by TrMab-6 and control mouse IgG_2b_ in CHO/TROP2, CHO-K1, MCF7, BINDS-29, MDA-MB-231, and MDA-MB-468 cells. The cells were incubated with 10% rabbit complement in the presence of the indicated antibodies for 5 h. Cytotoxicity (%) was calculated as described in Materials and methods. Values are mean ± SEM. Asterisk indicates statistical significance (**P<0.01, *P<0.05, n.s., not significant, Welch's t-test).

TrMab-6 was also associated with more robust CDC activity (67.7% cytotoxicity) in CHO/TROP2 cells compared to control mouse IgG_2b_ (33.9% cytotoxicity; P<0.05), in contrast to its CDC activity in CHO-K1 cells (Fig. 3). Furthermore, while TrMab-6 exhibited higher CDC (51.6% cytotoxicity) in MCF7 cells compared to the control (30.2% cytotoxicity; P<0.05), this was not evident in BINDS-29 cells (Fig. 3). TrMab-6 also exhibited higher CDC (36.0% cytotoxicity) in MDA-MB-231 cells than that of the control mouse IgG_2b_ (14.7% cytotoxicity; P<0.05) (Fig. 3). Furthermore, TrMab-6 exhibited higher CDC (47.0% cytotoxicity) in MDA-MB-468 cells than that of the control mouse IgG_2b_ (20.4% cytotoxicity; P<0.01) (Fig. 3).

These favorable ADCC/CDC activities indicated that TrMab-6 may induce strong antitumor activity against breast cancer cells *in vivo*.

#### Antitumor effect of TrMab-6 in mouse xenografts of TROP2-expressed CHO/TROP2 cells

**Figure 4.**
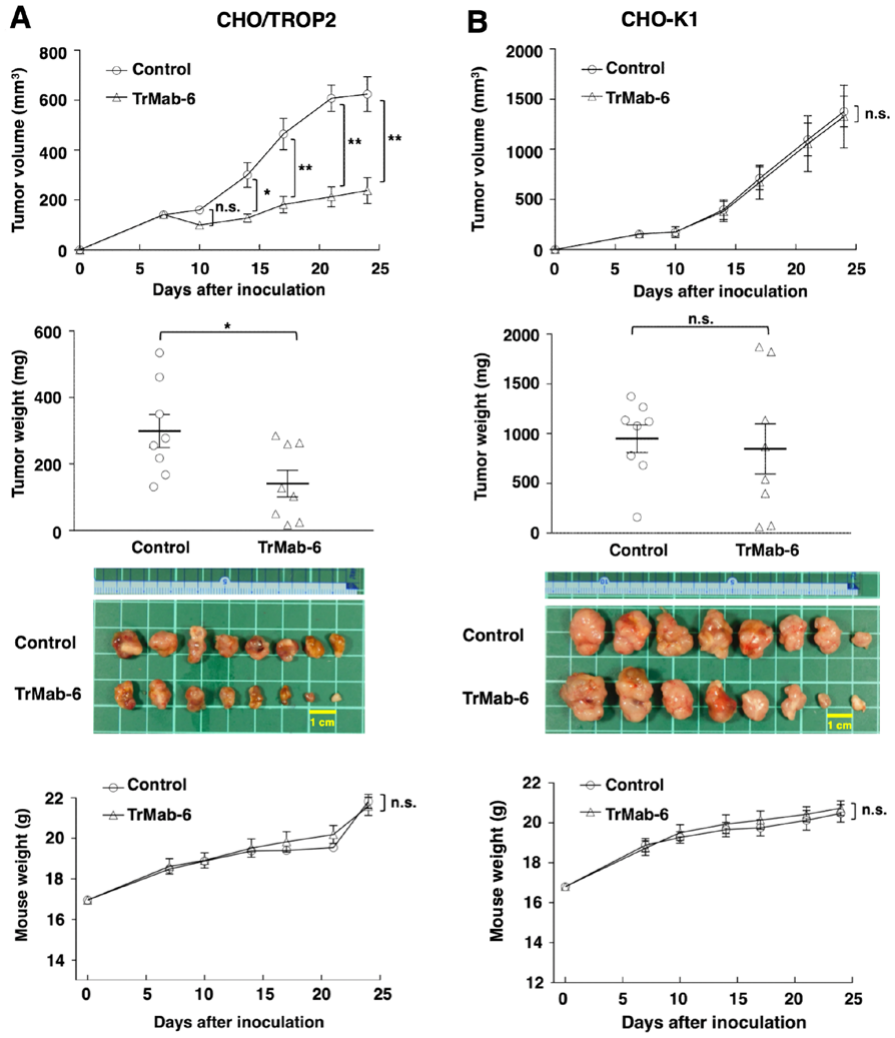
Evaluation of antitumor activity of TrMab-6 in CHO/TROP2 or CHO-K1 ×enografts. (A, upper panel) CHO/TROP2 cells (5×10^6^ cells) were injected subcutaneously into the left flank. After day 7, 100 µg of TrMab-6 and control mouse IgG in 100 µl PBS were injected i.p. into treated and control mice, respectively. Additional antibodies were then injected on days 14 and 21. The tumor volume was measured on days 7, 10, 14, 17, 21 and 24. Values are mean ± SEM. Asterisk indicates statistical significance (**P<0.01, *P<0.05, n.s., not significant, ANOVA and Sidak's multiple comparisons test). (A, middle panels) Tumors of CHO/TROP2 ×enografts were resected from TrMab-6 and control mouse IgG groups. Tumor weight on day 24 was measured from excised xenografts. Values are mean ± SEM. Asterisk indicates statistical significance (*P<0.05, Welch's t-test). Resected tumors of CHO/TROP2 ×enografts from control mouse IgG and TrMab-6 groups on day 24. Scale bar, 1 cm. (A, lower panel) Body weights of the mice implanted with CHO/TROP2 ×enografts were recorded on days 7, 10, 14, 17, 21 and 24 (n.s., not significant). (B, upper panel) CHO-K1 cells (5×10^6^ cells) were injected subcutaneously into the left flank. After day 7, 100 µg of TrMab-6 and control mouse IgG in 100 µl PBS were injected i.p. into treated and control mice, respectively. Additional antibodies were then injected on days 14 and 21. Tumor volume was measured on days 7, 10, 14, 17, 21 and 24. Values are mean ± SEM. n.s., not significant. (B, middle panels) Tumors of CHO-K1 ×enografts were resected from TrMab-6 and control mouse IgG groups. Tumor weight on day 24 was measured from excised xenografts. Values are mean ± SEM. n.s., not significant. Resected tumors of CHO-K1 ×enografts from control mouse IgG and TrMab-6 groups on day 24. Scale bar, 1 cm. (B, lower panel) Body weights of the mice implanted with CHO-K1 ×enografts were recorded on days 7, 10, 14, 17, 21 and 24 (n.s., not significant).

CHO/TROP2 was developed in our previous study (16). Tumor formation of 16 CHO/TROP2-bearing mice was observed on day 7. Then, these 16 CHO/TROP2-bearing mice were divided into a TrMab-6-treated group and a control group. On days 7, 14 and 21 after CHO/TROP2 cell injections into the mice, TrMab-6 (100 µg) or control mouse IgG (100 µg) were injected i.p. to the mice. Tumor volume was measured on days 7, 10, 14, 17, 21 and 24 after CHO/TROP2 cell injection. TrMab-6-treated mice exhibited significantly less tumor growth on days 14 (P<0.05), 17 (P<0.01), 21 (P<0.01), and 24 (P<0.01) compared with IgG-treated control mice (Fig. 4A, upper panel). On day 24, there was a reduction of the tumor volume of 61.9% in TrMab-6-treated mice (Fig. 4A, upper panel). Tumors from TrMab-6-treated mice weighed significantly less than tumors from IgG-treated control mice on day 24 (52.9% reduction, P<0.05; Fig. 4A, middle panels). These results indicated that TrMab-6 reduced the growth of CHO/TROP2 ×enografts, but without full elimination. Total body weights did not significantly differ between the treatment and control groups (Fig. 4A, lower panel).

Similarly, tumor formation of 16 CHO-K1-bearing mice was observed on day 7, before they were divided into a TrMab-6-treated group and a control group. On days 7, 14 and 21 after CHO-K1 cell injections, TrMab-6 (100 µg) or control mouse IgG (100 µg) were injected i.p. into the mice. Tumor volume was measured on days 7, 10, 14, 17, 21 and 24 after CHO-K1 cell injection. Both TrMab-6-treated and control groups exhibited similar tumor growth on all days (Fig. 4B, upper panel) and no difference in the tumor weight was observed between the two groups on day 24 (Fig. 4B, middle panels). These results indicated that TrMab-6 did not reduce the growth of TROP2-negative CHO-K1 ×enografts. Additionally, the total body weights did not significantly differ between the two study groups (Fig. 4B, lower panel).

#### Antitumor effect of TrMab-6 in mouse xenografts of TROP2-expressing MCF7 breast cancer cell lines

**Figure 5.**
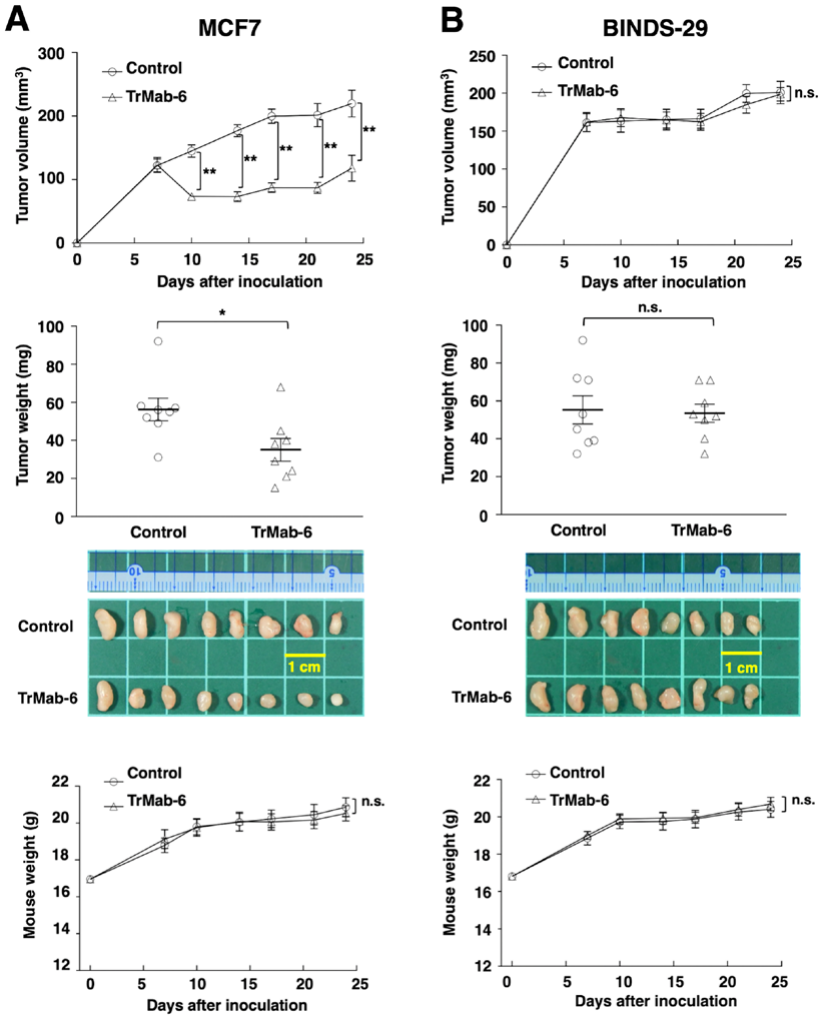
Evaluation of antitumor activity of TrMab-6 in MCF7 or BINDS-29 ×enografts. (A, upper panel) MCF7 cells (5×10^6^ cells) were injected subcutaneously into the left flank. After day 7, 100 µg of TrMab-6 and control mouse IgG in 100 µl PBS were injected i.p. into treated and control mice, respectively. Additional antibodies were then injected on days 14 and 21. Tumor volume was measured on days 7, 10, 14, 17, 21 and 24. Values are mean ± SEM. Asterisk indicates statistical significance (**P<0.01, ANOVA and Sidak's multiple comparisons test). (A, middle panels) Tumors of MCF7 ×enografts were resected from TrMab-6 and control mouse IgG groups. Tumor weight on day 24 was measured from excised xenografts. Values are mean ± SEM. Asterisk indicates statistical significance (*P<0.05, Welch's t-test). Resected tumors of MCF7 ×enografts from control mouse IgG and TrMab-6 groups on day 24. Scale bar, 1 cm. (A, lower panel) Body weights of the mice implanted with MCF7 ×enografts were recorded on days 7, 10, 14, 17, 21 and 24 (n.s., not significant). (B, upper panel) BINDS-29 cells (5×10^6^ cells) were injected subcutaneously into the left flank. After day 7, 100 µg of TrMab-6 and control mouse IgG in 100 µl PBS were injected i.p. into treated and control mice, respectively. Additional antibodies were then injected on days 14 and 21. Tumor volume was measured on days 7, 10, 14, 17, 21 and 24. Values are mean ± SEM. n.s., not significant. (B, middle panels) Tumors of BINDS-29 ×enografts were resected from TrMab-6 and control mouse IgG groups. Tumor weight on day 24 was measured from excised xenografts. Values are mean ± SEM. n.s., not significant. Resected tumors of BINDS-29 ×enografts from control mouse IgG and TrMab-6 groups on day 24. Scale bar, 1 cm. (B, lower panel) Body weights of the mice implanted with BINDS-29 ×enografts were recorded on days 7, 10, 14, 17, 21 and 24 (n.s., not significant).

The tumor formation of 16 MCF7-bearing mice was observed on day 7 before mice were divided into a TrMab-6-treated group and a control group. On days 7, 14 and 21 after MCF7 cell injections into the mice, either TrMab-6 (100 µg) or control mouse IgG (100 µg) was injected i.p. into the mice. The tumor volume was measured on days 7, 10, 14, 17, 21 and 24 after MCF7 cell injection. TrMab-6-treated mice exhibited significantly less tumor growth on days 10 (P<0.01), 14 (P<0.01), 17 (P<0.01), 21 (P<0.01), and 24 (P<0.01) compared with IgG-treated control mice (Fig. 5A, upper panel). On day 24, a reduction of the tumor volume of 46.6% was seen in TrMab-6-treated mice (Fig. 5A, upper panel). Tumors from TrMab-6-treated mice weighed significantly less than tumors from IgG-treated control mice on day 24 (37.8% reduction, P<0.05; Fig. 5A, middle panels). These results indicated that TrMab-6 reduced the growth of MCF7 ×enografts, but did not contribute towards their total elimination. Total body weights did not significantly differ between the treatment and control groups (Fig. 5A, lower panel).

Similarly, the tumor formation of 16 BINDS-29-bearing mice was observed on day 7, before the 16 BINDS-29-bearing mice were divided into a TrMab-6-treated group and a control group. On days 7, 14 and 21 after BINDS-29 cell injections into the mice, TrMab-6 (100 µg) or control mouse IgG (100 µg) was injected i.p. into the mice. The tumor volume was measured on days 7, 10, 14, 17, 21 and 24 after BINDS-29 cell injection. The TrMab-6-treated and control groups exhibited similar tumor growth on all days (not significant; Fig. 5B, upper panel) and no difference in the tumor weight was observed between the two groups, on day 24 (Fig. 5B, middle panels). These results indicated that TrMab-6 did not reduce the growth of TROP2-negative BINDS-29 ×enografts. Total body weights did not significantly differ between the treatment and control groups (Fig. 5B, lower panel).

## Discussion

TROP2 has been demonstrated to be overexpressed in a variety of tumors (18). A gene expression pattern analysis comparing gastric tumors and their normal counterparts revealed that TROP2 was not overexpressed in normal tissues (19). In addition, in a meta-analysis that included 16 studies involving 2,569 participants, TROP2 overexpression was found to be associated with poor overall and disease-free survival across several types of solid tumors (20). Furthermore, the knockdown of TROP2 decreased cell proliferation and migration (21). Altogether, these results suggest that TROP2 is a potential target for antitumor treatments.

Antibody-based therapy is a rapidly emerging field for treatment of several diseases, including cancer. The development of antibody drugs for TROP2 has been accelerated in recent years due to identification of the extracellular domain of TROP2 as a potential prominent target for TROP2-positive cancers (5,6). Among them, antibody-drug conjugates (ADCs) are the main modality of antibody drugs (22). Recently, the first anti-TROP2 ADC, sacituzumab govitecan, which is a humanized IgG_1_ conjugated to irinotecan metabolite (SN-38), has been approved by the US Food and Drug Administration against metastatic triple-negative breast cancers (23). ADCs have also been developed against hormone receptor-positive breast cancers and HER2-negative metastatic breast cancers (23). Preliminary findings have demonstrated that datopotamab deruxtecan (DS-1062) is active in patients with advanced or metastatic non-small cell lung cancer (24). In phase I trials of datopotamab deruxtecan, this drug induced responses in almost 25% of the patients trialed and had manageable side effects (24). The combination of these ADCs with immune checkpoint inhibitors is also expected to be effective (5,22,23,25).

The adaptive or acquired resistance to targeted antibody cancer therapies is of importance for clinical outcomes (26). Development of new antibodies and improvement of antibody-based drugs are required to overcome therapeutic resistance and reduce the possibility of identifying suitable candidates for clinical application (27). In this study, we demonstrated the efficacy of a new anti-TROP2 antibody, TrMab-6. The development of anti-TROP2 ADC is a potential therapeutic option for cancer patients with therapy-resistant solid tumors by itself or in combination with other anticancer drugs. Furthermore, TrMab-6 can be used in flow cytometry, immunohistochemistry, and western blot analyses (16). In histopathology, immunohistochemistry is used for clinical diagnosis for biopsies and resected specimens. TrMab-6 may be used to ascertain patients who should receive the anti-TROP2-targeted therapy.

The CBIS method, which uses antigen-expressing cell lines for both immunization and screening, can help to effectively develop mAbs which may be useful as antitumor agents. We have recently succeeded in developing numerous mAbs that target membrane proteins, including CD19 (28), CD20 (29), CD44 (30), CD133 (17), EpCAM (31), and TROP2 (16). Of these, CMab-43 (mouse IgG_2a_) for CD133 showed significant ADCC/CDC activities against colon cancer cells and antitumor activity against colon cancer xenograft models (32). EpMab-16 (mouse IgG_2a_) for EpCAM also demonstrated significant antitumor activity against colon cancer xenograft models (31), and oral squamous cell carcinomas (33). Furthermore, 5-mG_2a_-f (a defucosylated mouse IgG_2a_-type of clone C_44_Mab-5) for CD44 exerted antitumor effects in mouse xenograft models of oral squamous cell carcinomas (34).

In the present study, we investigated whether TrMab-6 (16), developed using the CBIS method, could exhibit ADCC/CDC activities *in vitro* and antitumor activity *in vivo* against breast cancers. *In vitro* experiments revealed strong ADCC/CDC inducement against CHO/TROP2, MCF7, MDA-MB-231, and MDA-MB-468 cells by TrMab-6 (Figs. 2 and 3). *In vivo* experiments on CHO/TROP2 (Fig. 4) and MCF7 (Fig. 5) xenografts revealed that the TrMab-6 treatment significantly reduced tumor growth, compared with the control mouse IgG. By contrast, TrMab-6 did not demonstrate ADCC/CDC *in vitro* (Figs. 2 and 3) and antitumor activity *in vivo* against TROP2-negative CHO-K1 (Fig. 4) and BINDS-29 (Fig. 5), demonstrating that the toxicity of TrMab-6 is specific for TROP2. These data indicated that TrMab-6 is a promising treatment option for TROP2-expressing breast cancers. For future studies, several modalities, such as ADC or chimeric antigen receptor (CAR)-T of TrMab-6, should be developed to strengthen the antitumor activity against breast cancers.

## Data Availability

The datasets used and/or analyzed during the study are available from the corresponding author on reasonable request.

## Abbreviations

ADC: antibody-drug conjugate
ADCC: antibody-dependent cellular cytotoxicity
BSA: bovine serum albumin
CBIS: Cell-Based Immunization and Screening
CDC: complement-dependent cytotoxicity
DMEM: Dulbecco's modified Eagle's medium
EDTA: ethylenediaminetetraacetic acid
FBS: fetal bovine serum
mAb: monoclonal antibody
PBS: phosphate-buffered saline
PVDF: polyvinylidene difluoride
SEM: standard error of the mean
